# Effects of Inhalation Aromatherapy on Symptoms of Sleep Disturbance in the Elderly with Dementia

**DOI:** 10.1155/2017/1902807

**Published:** 2017-03-19

**Authors:** Ai Takeda, Emiko Watanuki, Sachiyo Koyama

**Affiliations:** ^1^Department of Nursing, Tokyo Ariake University of Medical and Health Sciences, 2-9-1 Ariake, Koto-ku, Tokyo 135-0063, Japan; ^2^Kitasato University School of Nursing, 2-1-1 Kitasato, Minami-ku, Sagamihara, Kanagawa 252-0329, Japan

## Abstract

This study investigated the effects of inhalation aromatherapy on sleep disturbance in elderly individuals with dementia. In 19 subjects, normal sleep was observed for a 20-day control period, inhalation aromatherapy was then applied for a 20-day intervention period, and the control and intervention periods were compared. During the intervention period, essential oils were placed nightly on towels around the subjects' pillows. The measured sleep conditions were sleep latency, total sleep time, sleep efficacy, duration of the longest sustained sleep period, wake time after sleep onset, early morning awakening, total daytime sleep, and the Neuropsychiatric Inventory. Total sleep time was significantly longer in the intervention period than in the control period (*p* < 0.05). The duration of the longest sustained sleep period was significantly longer in the intervention period than in the control period (*p* < 0.05). Early morning awakening in the intervention period was significantly less compared to that in the control period (*p* < 0.05). Total daytime sleep could not be adequately measured and was omitted from the analysis. No significant differences in other sleep conditions were observed. These results indicated positive effects of inhalation aromatherapy on symptoms of sleep disturbance in elderly individuals with dementia.

## 1. Introduction

Japan is facing the dual challenges of an increasingly aging population and a greater number of elderly persons with dementia [1]. Aging is associated with several well-described changes in the sleep pattern. In addition, sleep disturbance or disruption is common among patients with dementia [2]. Older adults with insomnia have been found to show a pattern of increased activation of subcortical brain areas during sleep; therefore, they often feel tired during the daytime. On the other hand, diminution of sleep time has been found to decrease prefrontal cortical activation during wakefulness, which also causes sleep problems. Sleep disturbance is intricately entwined with sense of well-being, health, emotion regulation, performance and productivity, memory and cognitive functions, and social interaction [3]. Methods of promoting nocturnal sleep in the elderly with dementia are therefore needed.

Causes for the onset of sleep disturbance in the elderly with dementia include psychological and physical factors, such as loneliness, anxiety, pain, incontinence, hunger, and constipation; ways in which care staff deal with them; and environmental factors, such as noise, light, and temperature. Aging and organic changes in the brain are also involved. Assessment of these discomforts is expressed through sleep disturbance, and addressing the underlying discomfort is both necessary and effective [4]. However, accurate assessment and identification of the cause of sleep disturbance in the elderly with dementia are often extremely difficult. Among elderly, individuals with dementia in Japan who currently suffer from sleep disturbance receive prescriptions for anxiolytics or other medications, indicating that resolving sleep disturbance through pharmacotherapy takes priority over approaches that target cause. However, administration of antipsychotic or anxiolytic medication for the purpose of inducing sleep increases mortality rate [5], in many cases, can aggravate physical and psychological symptoms, and can potentially lead to falls, dysphagia, and other problems. For this reason, there is great interest in nonpharmacological treatment strategies in order to reassess the present pharmacotherapy-centered approach.

Interest has been growing in the sleep-promoting effects of aromatherapy, which is a nonpharmacological treatment. Aromatherapy is a natural treatment method that uses essential oils extracted from aromatic plants; these essential oils are believed to have medicinal actions and to influence the brain, mind, and body [6]. Aromatherapy may therefore facilitate improvement in sleep in elderly individuals with dementia, regardless of the various sleep disturbance symptoms and causes. Moreover, as essential oils require no invasive administration modalities, aromatherapy offers the advantages of minimal recipient burden and easy administration by staff.

Although the precise mechanisms by which the essential oils used in aromatherapy promote sleep are not fully known, sleep-promoting and sedative effects have been verified. Tanida et al. [7] carried out experiments on rats and showed that the lavender aroma and the linalool component inhibit sympathetic nervous system activity and heighten parasympathetic nervous system activity.Heuberger et al. [8] reported that linalool, which is the main component of lavender, shows sedative actions in healthy adults. Ohmori et al. [9] reported that an essential oil containing santalol, which is present in sources such as sandalwood, significantly improves total waking time and NREM sleep in sleep-disturbed rats. Yamamoto et al. [10] showed that nonfragrant lavender and cedrol, which is a component of cedar wood essential oil, improve sleep in young women. Yamagishi et al. [11] reported that piperonal, which is extracted from plants of the laurel family, promotes sleep in healthy adults. The mechanism of action involves integration of the essential oil into a biological signal for olfactory receptors during inhalation. This signal is transmitted to the limbic and hypothalamic areas of the brain via the olfactory bulb. Such signals cause the brain to release neurotransmitters, such as serotonin and endorphins, that link the nervous system to other body systems and provide a desired change and feeling of relief. Release of serotonin, endorphins, and noradrenalin is achieved with calming, euphoric, and stimulating oils, respectively, to give predictable effects on the mind and body [12].

While these reports have found that aromatherapy has sleep-promoting effects, very few studies have examined the use of aromatherapy for sleep promotion in elderly individuals with dementia. Imanishi et al. [13] provided aromatherapy massage for elderly individuals with disturbed sleep who were residents at a care facility for the elderly and used actigraphy to compare sleep before and after massage. They reported significant increases in sleep rate and sleep efficacy (SE), suggesting that the intervention did have an effect on sleep. However, the extent to which those results represented the effects of massage or aromatherapy could not be determined. Therefore, while case reports have described improved sleep among sleep-disturbed elderly with dementia [14, 15], statistical analysis with multiple subjects has only been performed in one study [13]. Whether sleep can be promoted from inhalation of the aroma alone as opposed to application of the essential oil to the skin also needs to be verified.

Based on the findings of previous studies that the aroma components linalool, santalol, cedrol, and piperonal promote sleep by heightening parasympathetic activity, in the present study, the researchers hypothesized that administration of inhalation aromatherapy to sleep-disturbed elderly with dementia would result in improvement in sleep disturbance.

## 2. Methods

### 2.1. Subjects and Method of Selection

Nurses working at three care facilities for the elderly in the same prefecture were provided detailed explanations on the operational definition of sleep disturbance. Elderly individuals with dementia who were ≥65 years old and who were diagnosed with sleep disturbance by the nurses were selected as potential subjects. Consent was obtained from 22 elderly individuals and their families (guardians), who then became the study subjects. However, individuals who had commenced or altered use of sleep medication, anxiolytic medication, antipsychotic medication, or steroids (hereafter, collectively referred to as medications with soporific effect) in the 2 weeks prior to the selection date, individuals undergoing treatment for sleep apnea syndrome, and individuals with sleep disturbance resulting from physical symptoms such as pain were excluded.

### 2.2. Operational Definitions of Terms

In the present study, “sleep disturbance” of elderly persons with dementia was operationally defined on the basis of the general diagnostic standards of the International Classification of Sleep Disorders, Revised (ICSD-2) [16]:

(A) One or more of symptoms (1)–(3) are observed and have persisted for at least the previous 3 months. (1) Difficulty initiating sleep: 30 min or more required to initiate sleep after going to bed. (2) Difficulty maintaining sleep: waking three or more times during sleep, each time requiring 30 min or more to reinitiate sleep. (3) Early morning awakening (EMA): waking before 03:00 with inability to reinitiate sleep.
(B) Symptoms in (A) occur even though the opportunities for sleep and the sleep environment are adequate.
(C) (A) and (B) both apply, and either or both daytime sleep or behavioral and psychological symptoms in dementia (BPSD) are present.

### 2.3. Study Methods

Three types of essential oil were used for aromatherapy: true lavender, true lavender and sweet orange oil blend (Re:Brain; HyperBrain, Tottori), and Japanese cypress, Virginian cedarwood, cypress, and pine oil blend (Relaxation Woody; HyperBrain, Tottori). Each of these three types is 100% essential oil and contains two or more of linalool, santalol, cedrol, and piperonal. Before the start of the study, the subjects themselves selected one oil from among the three to be used in the study.

The first 20 days were set as the control period and the second 20 days as the intervention period. During the control period, a researcher visited each subject in his or her room between 18:00 and 20:00 every day and wrapped a towel with nothing on it around the subject's pillow before the subject went to bed. During the intervention period, a researcher visited each subject in his or her room at the same time and wrapped a towel with essential oil on it around the subject's pillow before the subject went to bed. The essential oil was the one chosen by the subject, and the researcher prepared the towel by asking the subject for the preferred amount of oil in the range of 2–5 drops (0.1–0.25 ml). During both the control period and the intervention period, the towel was collected the following morning. In order to avoid the Hawthorne effect, the researcher ensured the same environments in the control and intervention periods. In addition, during both the control and intervention periods, the researcher asked the subject about sleeping and health status and observed the subject, while ensuring that the environment remained unchanged.

### 2.4. Measurements and Data Collection

Data on age, gender, length of residency, severity of dementia, activities of daily living (ADL), and use of medication with soporific effects were collected. Dementia severity was measured using the mini-mental state (MMS) [17], which was administered to the subjects before the start of the study. MMS comprises 11 items, including orientation, recall, and calculation, which are scored to a total of 0–30 points, with a lower score indicating greater dementia severity. ADL was measured using the Functional Independence Measure (FIM) [18]. The FIM comprises 13 mobility ADL items, including eating and transfer, and five cognitive ADL items. Only the mobility items were used in the present study. Each item is scored from 1 to 7, giving a score in the range 13–91. A higher score indicates greater independence in ADL.

To ascertain whether the sleep environment was appropriate, data on the times of going to bed and rising were collected every day during the study as environmental characteristics. Time of going to bed was the first time that the subject went to bed after 18:00, while time of rising was the last time that the subject rose before 08:00. Data were collected using a sheet-type body vibrometer, as described below. The nursing staff at each facility was interviewed to check that. Other than the data collected by the vibrometer, no changes in room temperature, room environment, care system, and so forth occurred during the study period.

Using the vibrometer, 24 h measurements were taken for 40 days, and sleep disturbance symptoms were measured as follows:

- Difficulty initiating sleep: sleep latency (SL)
- Difficulty maintaining sleep: total sleep time (TST), SE, duration of longest sustained sleep period (DLSSP), and wake time after sleep onset (WTASO)
- EMA: number of times of early morning awakening
- Daytime disorder: BPSD and daytime sleep

BPSD was measured using the Neuropsychiatric Inventory (NPI), which is a comprehensive scale of psychiatric symptoms developed by Cummings et al. [19]; in this, subjects are evaluated on 10 items (delusions, agitation, hallucinations, irritability, depression, anxiety, euphoria, apathy, disinhibition, and aberrant motor behavior). Scores are in the range 0–120 and higher scores indicate greater BPSD severity. Permission to use this scale was granted by the translator, Dr. Nobutsugu Hirono, and the nursing staff at each facility was requested to carry out measurements once a week.

A sheet-type body vibrometer was used (Nemuri SCAN; Paramount Bed Co., Tokyo). This was placed under the mattress so as not to affect or hinder the sleep environment and was used to measure and record the time spent in bed, time of going to bed, time of rising, sleep time, waking time, and time spent out of bed during a 24 h period. Comparison of Nemuri SCAN data to data from an actinography device fitted to the arm, as used in prior studies, or data from a sleep polygraph, as used in sleep research worldwide, allows changes in sleeping and waking to be recorded with a high concordance rate [20]. In the present study, to distinguish between nocturnal and daytime sleep, sleep between 08:00 and 18:00 was designated as daytime sleep.

### 2.5. Statistical Analysis

Subject attributes and sleep indices were calculated using descriptive statistics. The environmental attributes of the time of going to bed, time of rising, and time spent in bed were compared between the control and intervention periods using a *t*-test. In addition, nursing staff were interviewed to determine whether there had been any changes in room temperature or number of staff on duty, or any sudden changes in other individuals in the room. Sleep disturbance in the control and intervention periods was then compared using two-way analysis of variance (ANOVA) and the Wilcoxon signed rank test. The software used was SPSS Statistics version 23.0 (IBM-Japan, Tokyo).

### 2.6. Ethical Considerations

The implementation of this study was approved by the research ethics committee of the authors' institution. After the manager of each facility provided consent, the subjects and their families/guardians were given oral and written explanations of the aims and nature of the research and were informed that consent could be withdrawn at any time. Where possible, subjects filled in the consent form themselves, and those subjects who had difficulty writing gave only verbal consent. In addition, families (guardians) gave consent by completing the consent form.

One member of the nursing staff in each facility was requested to cooperate with the study and to act as the point of contact between subjects, families, other nursing staff, and researchers. The study was to stop immediately if there were any changes or signs of discomfort, and families and all facility staff were requested to observe the subjects and report anything untoward. A researcher also visited every subject at about the same time every day to greet, observe, and ensure that there were no changes or signs of discomfort.

The intervention was only carried out by the researcher from this study, that is, a qualified nurse who had completed Level 3, the highest level of the Diploma in Holistic Therapies course as recognized by the Vocational Training Charitable Trust of the United Kingdom.

## 3. **Results**

### 3.1. General Characteristics of the Participants

Twenty-two elderly individuals and their families (guardians) consented to participate, of whom one person was hospitalized during the study period, one person wished to withdraw from the study during the control period, and one person commenced use of sleep medication during the study period. These three individuals were excluded, and the remaining 19 persons were the study subjects. The basic attributes of the 19 subjects are shown in Table 1.

### 3.2. Environmental Attributes

The mean time of going to bed for all subjects was 19:26 ± 01:16 during the control period and 19:24 ± 01:11 during the intervention period, with no significant difference between periods [*t*(18) = 0.39, *p* = 0.699]. The mean time of rising was 05:31 ± 01:15 during the control period and 05:41 ± 01:25 during the intervention period, with no significant difference [*t*(18) = −1.96, *p* = 0.066].

With regard to the temperature within the facilities and rooms where the subjects slept, the study took place from early summer to full summer and the temperature was set at 27°C for 24 h a day in each of the three facilities. In addition, no changes were seen in the arrangement of furniture or the personal belongings of the subjects in their rooms, nor were there any sudden changes in the other individuals sharing the rooms. With regard to the care system, the number of staff at work during the day, during the night, on the late shift, and on the early shift was checked, and no differences were apparent during the control and intervention periods. Therefore, the physical and human environment did not change and was not considered to influence the effects of the intervention, which were therefore verified without excluding certain days or performing covariance adjustment.

### 3.3. Changes in Sleep Disturbance

One subject took a drug with a soporific effect (e.g., psychotropic drug) that had been prescribed for use when necessary on one day during the control period and one day during the intervention period; for this subject, only those days on which the medication was used were excluded from the analysis. Three subjects had days during which they spent the whole day in bed due to slight fever; for these subjects, these days were excluded from the analysis. One subject complained of itching while in bed; for this subject, 4 days during which the subject complained of itching were excluded. These excluded days were treated as missing data. Because less than 50% of data were missing in all cases, the missing data were input by substitution of the mean case value [21] for the analysis.

### 3.4. Difficulty Initiating Sleep

Table 2 shows descriptive statistics and comparison of means for sleep disturbance symptoms between the control and intervention periods. Mean SL was 32.2 ± 17.8 min in the control period and 29.2 ± 17.7 min in the intervention period. Comparison by two-way ANOVA showed no significant differences for the main effect of the control and intervention periods, the main effect of time, or period × time.

### 3.5. Difficulty Maintaining Sleep

Mean TST was 444.3 ± 89.3 min during the control period and 461.2 ± 95.2 min during the intervention period, with a significant improvement seen with the main effect of the control and intervention periods [*F*(1) = 10.08, *p* = 0.005]. However, no significant difference was seen with the main effect of time or period × time.

Mean SE was 72.9 ± 11.3% during the control period and 74.6 ± 12.0% during the intervention period, with no significant difference seen with the main effect of the control and intervention periods, the main effect of time, or period × time.

Mean DLSSP was 173.0 ± 52.1 min during the control period and 186.2 ± 62.6 min during the intervention period. The results of two-way ANOVA showed a significant improvement with the main effect of the control and intervention periods [*F*(1) = 4.46, *p* = 0.049]. However, no significant difference was seen with the main effect of time or period × time.

Mean WTASO was 129.6 ± 59.8 min during the control period and 125.8 ± 70.0 min during the intervention period. No significant difference was seen with the main effect of the control and intervention periods, the main effect of time, or period × time.

### 3.6. EMA

Mean EMA was 1.5 ± 2.5 times during the control period (range: 0–10 times). Mean EMA was 0.7 ± 1.5 times during the intervention period (range: 0–6 times). The results of Wilcoxon's signed rank test were *z* = −2.59, *p* = 0.010, indicating a significant difference between the control and intervention periods.

### 3.7. Daytime Disorder

Daytime sleep time was taken only as time spent napping on the bed. However, daytime sleep data could not be collected at all in eight subjects, because although they were seen to have daytime naps while seated in their wheelchairs almost every day, they refused to stay in bed when encouraged to do so. Daytime sleep was therefore excluded as a study variable.

Mean NPI score was 6.4 ± 6.0 during the control period and 5.6 ± 6.0 during the intervention period. Two-way ANOVA indicated no significant difference with the main effect of the control period and intervention period or the main effect of time, while a significant difference was seen for period × time [*F*(1) = 5.41, *p* = 0.032].

## 4. Discussion

### 4.1. Effect of Inhalation Aromatherapy to Promote Sleep in the Elderly with Dementia

This study investigated the hypothesis that administering inhalation aromatherapy would improve sleep disturbance symptoms in the elderly with dementia. The results suggest that administration of inhalation aromatherapy may improve difficulties in maintaining sleep and EMA; that is, there was partial verification of the hypothesis. Because the influence of environmental attributes was checked and steps were taken to avoid the Hawthorne effect, improvement in sleep disturbance symptoms was attributed to the administration of inhalation aromatherapy.

We conjecture that the improvements seen in this study in sleep time, DLSSP, and EMA of elderly persons with dementia were because the various fragrance components of inhalation aromatherapy were taken up by the subjects and promoted secretion of serotonin and endorphins, resulting in activation of the parasympathetic nervous system. Endorphins have sedative effects, while serotonin binds to enzymes during the night to produce melatonin, thereby promoting sleep [22]. In the elderly, melatonin secretion declines with aging, causing biological rhythms to advance [22]. Facilitation of endorphin and serotonin secretion as a result of aromatherapy administration before going to bed is therefore likely to mean that even at the normal time of EMA, high concentrations of endorphins and melatonin remain in the blood. This would result in an increase in the DLSSP and would also reduce EMA because even if the elderly woke up in the early morning, they would be able to go back to sleep smoothly. This was probably the cause of the increase in sleep time. Brain waves were not studied with polysomnography and hormone levels in saliva and blood were not measured in order to minimize burden on the subjects who were elderly individuals with dementia. Further research is thus needed in these areas.

The significant increase in sleep time found in the present study agrees with similar results in young, healthy adults using cedrol [10] and piperonal [11]. Among the indices of difficulty in maintaining sleep, no effect was seen in the present study for SE or WTASO. Increased sleep time and DLSSP were expected to lead to decreased WTASO; however, WTASO did not decrease. Looking at the control period, the symptom of waking after sleep onset was seen in all study subjects. Deep sleep (stages 3-4) and REM sleep time have also been reported to decrease and waking after sleep onset increases in healthy elderly individuals [23] and subjects with Alzheimer-type dementia in comparison to healthy subjects of the same age [24]. Waking after sleep onset is a major issue affecting the sleep of many elderly people and individuals with dementia, and inhalation aromatherapy alone was unable to improve WTASO in these subjects. However, in a study with piperonal [11], REM sleep time and stage 4 sleep time of subjects increased significantly, while an increase in DLSSP was seen in the present study. Limiting the type of fragrance components may thus yield improvement. In addition, subjects in the present study showed a tendency toward improved SE, although no significant difference was found. Subjects in prior studies [10, 11] have also shown a tendency toward improvement in SE, without showing significant differences, which is in agreement with the present results. Even though sleep time increased, waking after sleep onset did not decrease, and there was hardly any increase in SE as a result.

SL, as an index for difficulty in initiating sleep in the present study, showed no significant difference between the control and intervention periods. This differs from the findings of prior studies in which SL decreased significantly as a result of aromatherapy in healthy women with a mean age of 25 years [10] and healthy adults aged 18–25 years [11]. Young subjects in these prior studies were recruited regardless of whether they had sleep disturbance symptoms; therefore, these groups probably did not have a long SL to start with. Even if SL can be further shortened in groups with no SL problems, SL seems unlikely to be improved by the effects of fragrant components or aroma as much as in groups in which SL is lengthened due to some sort of disorder. This will require further study.

Daytime sleep, as an index of daytime disorder, could not be accurately measured. For this reason, daytime disorder was evaluated only by NPI score, indicating the severity of BPSD. While improvement in sleep disturbance symptoms might be expected to be accompanied by improvements in other BPSD that have their origins in sleep disturbance, no improvement in NPI score, which indicates the level of BPSD, was seen among the present subjects. Jimbo et al. [25] administered inhalation aromatherapy to elderly subjects with Alzheimer-type dementia and examined changes in cognitive function scores. They administered inhalation aromatherapy to promote wakefulness during the daytime and tranquility at night for 24 h and reported significant improvements in cognitive function scores. The present study only applied inhalation aromatherapy at night because of the focus on sleep disturbance symptoms. Jimbo et al. [25] also reported that cognitive function improved as a result of aromatherapy rather than as a result of improvement in sleep disturbance, which may explain why the results of the present study were not in agreement. As daytime sleep data were not able to be collected in the present study, investigation into the relationship between daytime and nighttime sleeping is needed.

### 4.2. Implications for Nursing Practice and Study Limitations

The present study suggests the possibility that inhalation aromatherapy can improve difficulties in maintaining sleep and EMA in elderly persons with dementia. The sleep-wake pattern often seen in elderly persons with dementia is waking after sleep onset, then being unable to get back to sleep and as a result remaining awake from early morning onward. The effect that may be obtained from aromatherapy with such patients in particular is increase in the interval between waking after sleep onset and reinitiation of sleep even after awakening in the early morning, thus increasing overall sleep time. To obtain these effects, essential oil containing piperonal, santalol, linalool, and cedrol should be selected, and the aromatherapy should be continued for a period of at least approximately 20 days. The person administering aromatherapy must have sufficient knowledge on safety and should visit the patient's room in the evening and morning to carry out the tasks of wrapping and unwrapping the towel. Nonetheless, these tasks certainly seem to be worthwhile as a substitute for administering and managing medication. Furthermore, an advantage of this method is that it can be performed simply with a towel and essential oil without the need for devices to diffuse the aroma. This means that it is perfectly feasible to perform even for family caregivers or staff in busy clinical settings. We, therefore, intend to progress with the verification of aromatherapy in order to establish and expand this nonpharmacological treatment to improve sleep disturbances.

A limitation of this study is that it was impossible to grasp fully and examine the 24 h sleep-wake pattern because data on daytime sleep could not be accurately collected. An issue that arose during the present study was which index to use for identifying hindrances to normal daytime activities. This study aimed to evaluate daytime sleep and NPI; however, a problem with the Nemuri SCAN was that, as Kogure et al. [20] noted, sleep-wake patterns cannot be ascertained away from the bed. In fact, patients do not have daytime naps only on their beds but may also nap at mealtimes and during rehabilitation or recreation. Methods of measuring daytime sleep are therefore needed along with variables that are more appropriate than napping.

The small sample size should be noted as a limitation. Although the results suggested effects on EMA, examination of the effect on EMA adjusted for the influence of other factors was not possible because EMA did not show a normal distribution and the sample was too small to adjust for other factors. Rather than the number of times of EMA, a way to change this to a variable with a normal distribution is needed. In addition, it was not possible to inspect the differences in effect appearances among the essential oils. The three types of essential oils used in this study may improve sleep disturbance; further studies with a larger number of subjects should be conducted to compare these oils with respect to improvement in sleep disturbance. Furthermore, each essential oil's constituents need to be elucidated using chemical analyses before subjects receive aromatherapy intervention.

Alternatively, more elderly individuals with dementia suffering from sleep disturbance will have to be secured as subjects. Random selection of subjects was not possible, which probably biased the sample toward a greater number of individuals with low cognitive function scores and more severe disorders. The relevance of cognitive function score and the effect on sleep improvement were not verified because of this bias, and further study is needed.

Repeated investigation will need to be carried out to (1) determine whether the number of times and duration of waking after sleep onset can be improved among the elderly with dementia if the aromatherapy is limited to piperonal, (2) determine a method for measuring daytime dysfunction, and (3) explore the mechanism of the effect of inhalation aromatherapy without burden to the elderly with dementia.

## 5. Conclusion

In conclusion, the present study suggests that inhalation aromatherapy containing any of piperonal, santalol, linalool, or cedrol may improve difficulties in maintaining sleep and EMA in elderly persons with dementia. These findings indicate that it is worth repeating the verification of inhalation aromatherapy in sleep promotion for the elderly with dementia.

## Figures and Tables

**Table 1:** Sociodemographic characteristics of the 19 elderly individuals.

Variable	M ± SD	*n* (%)
Sex		
Male		10 (52.6)
Female		9 (47.4)
Age (years)	80.7 ± 9.1	
MMS	6.0 ± 6.5	
FIM	39.1 ± 24.1	
Length of stay (months)	19.1 ± 19.1	

MMS, mini-mental state; FIM, functional independence measure.

*N* = 19.

**Table 2:** Means and comparisons of sleep indices between the control period and the intervention period.

Variable	Control period		Intervention period		Period^b^		Time		Period × time	
	M	SD	M	SD	*F* ^c^	*p*	*F* ^c^	*p*	*F* ^c^	*p*
SL (min)	32.2	17.8	29.2	17.7	0.75	0.399	1.19	0.261	0.85	0.649
TST (min)	444.3	89.3	461.2	95.2	10.08	0.005^*∗∗*^	0.99	0.471	0.98	0.483
SE (%)	72.9	11.3	74.6	12.0	4.26	0.054	0.95	0.519	1.02	0.435
DLSSP (min)	173.0	52.1	186.2	62.6	4.46	0.049^*∗*^	1.33	0.159	0.76	0.754
WTASO (min)	129.6	59.8	125.8	70.0	0.31	0.586	0.59	0.914	0.75	0.767
EMA (times)^a^	1.5	2.5	0.7	1.5	−2.59	0.010^*∗*^				
NPI	6.4	6.0	5.6	6.0	3.86	0.065	2.67	0.083	5.41	0.032^*∗*^

*Note*. ^a^*Z*-value refers to overall time of each period effect using Wilcoxon signed rank test.

^b^Period comparing the control and intervention periods.

^c^ *F*-value refers to main and alternate effects using two-way ANOVA.

*N *= 19.

^*∗*^ *p* < 0.05, ^*∗∗*^*p* < 0.01.
